# Real-Life Use of Tocilizumab in the Treatment of Severe COVID-19 Pneumonia

**DOI:** 10.1155/2022/7060466

**Published:** 2022-06-09

**Authors:** Ruth Alex, Shabaz Mohiuddin Gulam, Kiran Kumar

**Affiliations:** ^1^College of Pharmacy, Gulf Medical University, Ajman, UAE; ^2^Dept. of Pharmacy Practice, College of Pharmacy, Gulf Medical University, Ajman, UAE; ^3^Dept. of Clinical Pharmacy, Thumbay University Hospital, Ajman, UAE; ^4^Dept. of Internal Medicine, Thumbay Hospital, Ajman, UAE; ^5^College of Medicine, Gulf Medical University, Ajman, UAE

## Abstract

**Introduction:**

Coronavirus disease 2019 (COVID-19) can progress to severe respiratory compromise and lead to mortality due to induction of cytokine storm. Tocilizumab (TCZ) is approved by the FDA for the treatment of cytokine release syndrome (CRS). This study aims to analyze the outcomes among patients who received TCZ in the United Arab Emirates.

**Methods:**

A retrospective cohort study was conducted among COVID-19 patients who received TCZ in a tertiary care hospital from May 2020 to August 2021. For analysis, patients were divided into two groups based on survival and clinical improvement.

**Results:**

Overall, 80% of patients receiving TCZ were discharged by day 28. There was a gradual improvement in oxygen requirements in our patients with a majority of them on room air by day 28. Age more than 50 years (*P*=0.034) and comorbidities such as cardiovascular disease (CVD) (*P*=0.002) and renal insufficiency (*P*=0.013) were significantly associated with mortality. *Discussion*. In our analysis, patients who were mechanically ventilated at the time of administration of TCZ had a significantly higher risk of death by day 28. In both survived and improved groups, younger patients had better outcomes than older patients. Patients who received TCZ earlier during therapy from the onset of symptoms had better survival outcomes. There was only one death among 14 patients who received vaccination. There was no significant difference in mortality among patients with comorbidities such as diabetes, hypertension, dyslipidemia, obesity, and pulmonary diseases, hypothesizing that administration of TCZ improves the outcomes in COVID-19 patients with these comorbidities.

## 1. Introduction

The novel coronavirus disease (COVID-19) is an unparalleled international public health crisis and has majorly impacted the global economy and healthcare system all over the world [1]. Since the United Arab Emirates (UAE) is a cosmopolitan country and acts as a crossroad for global economy and trade, the COVID-19 infection surged quickly compared to other countries in the region [2, 3]. The first confirmed case of COVID-19, caused by severe acute respiratory syndrome coronavirus 2 (SARS-CoV-2), in the UAE was announced on 29 January 2020 [4]. UAE was the first country in the Middle East to report a confirmed case [4]. The first two deaths in the UAE were reported on 20 March 2020 [5].

The UAE government rapidly responded with swift and decisive public health measures beginning with initiating an emergency response system, providing guidance for risk communication with the public, building field hospitals and screening facilities. The authorities also provided regular and updated scientific recommendations for the clinical management of this disease [6–8].

The National Guidelines for Clinical Management and Treatment of COVID-19, published on 19 March 2020, provided a protocol on the practical steps to deal with COVID-19 cases. Recommended therapy for patients with severe pneumonia or critical illness included lopinavir-ritonavir, chloroquine or hydroxychloroquine, and favipiravir with or without pegylated interferon [7]. An update of the National Guidelines, published on 3 April 2020, had significant changes with a recommendation on the use of tocilizumab (TCZ) if the patient is in early acute respiratory distress syndrome (ARDS) and possible cytokine storm [8]. These guidelines are continuously updated with the latest evidence-based scientific information [7, 8].

TCZ is a humanized monoclonal antibody that acts as an interleukin 6 (IL-6) receptor inhibitor and is approved by the FDA for the management of cytokine release syndrome (CRS) that is caused by chimeric antigen receptor T-cell (CAR-T) therapy [9]. The involvement of CRS in COVID-19 worsening is demonstrated by the increased proinflammatory factors that are seen in severe COVID-19 patients [9]. Studies report varying success rates of TCZ in patients with COVID-19 [10–14]. Predictors of poor outcome in COVID-19 patients receiving TCZ, according to studies conducted globally, include older age, comorbidities such as hypertension, diabetes mellitus, and immunosuppression, days from diagnosis until TCZ administration, elevated C-reactive protein (CRP), lactate dehydrogenase (LDH), troponin I, and neutrophil levels [15–17]. This study aims to analyze the baseline risk factors of survival and clinical improvement among patients who received TCZ hospitalized in a tertiary care hospital in the UAE.

## 2. Methods

### 2.1. Design

This is a retrospective cohort study conducted among patients admitted at Thumbay Hospital, Ajman for COVID-19 pneumonia between May 2020 and August 2021. Patients, who were severely ill with extensive lung disease, and with high levels or progressively increasing levels of D-dimer or CRP or ferritin, and worsening of respiratory exchanges received TCZ.

Clinical features, comorbidities, laboratory investigations, oxygen requirements, and treatment details of all patients included in the analysis were recorded. The data were obtained from electronic medical records after approval by the Institutional Ethical Committee. Written consent for compassionate use of TCZ was obtained from the patient or relatives by the primary care team before administration.

### 2.2. Treatment

TCZ was administered intravenously at a dose of 8 mg/kg, with a maximum first dose of 800 mg. A second dose of 400 or 600 mg was given in case of laboratory or respiratory worsening with a maximum cumulative dose of 1400 mg. Patients also received supportive therapy when required with supplemental oxygen, steroids, antivirals, antibiotics, vasopressors, renal replacement therapy, and other supplemental treatments as determined by the primary team.

### 2.3. Outcomes

Patients' clinical status was assessed using a six-category ordinal scale, similar to previously published studies of COVID-19 (modified from the WHO guidelines), on Day 1, Day 14, and Day 28 [18–21].

The categories include (1) patient discharged, (2) hospitalization, not requiring supplemental oxygen, (3) hospitalization requiring supplemental low-flow oxygen, (4) hospitalization requiring high-flow supplemental oxygen and/or noninvasive ventilation (NIV), (5) hospitalization requiring invasive mechanical ventilation (MV) or extracorporeal membrane oxygenation (ECMO), and (6) death.

Clinical improvement was defined as a two‐point reduction in patient's baseline status on the six‐point ordinal scale or live discharge from the hospital, whichever came first on Day 28. Predictors of survival and clinical improvement were analyzed in TCZ patients.

### 2.4. Oxygen Requirement

Supplemental oxygen is categorized as follows: Low-flow oxygen—nasal prongs (NP) 2–4 L, face mask (FM) 5–10 L, and nonrebreather mask (NRBM) 8–15 L. High-flow oxygen—noninvasive ventilation (NIV) alternating FM 5–10 L, NIV alternating NRBM 10–15 L, NIV alone, and high-flow nasal cannula (HFNC). Mechanical ventilation was provided to patients who required intubation.

### 2.5. Statistical Analyses

Data were analyzed using SPSS version 27.0 (SPSS, Chicago, IL, USA). Continuous variables were analyzed for significance using the independent *t*-test, and categorical variables were analyzed using chi-square. Survival analysis was performed with the Kaplan–Meier approach, and the log-rank test was used to compare survival curves. *P* values <0.05 were considered statistically significant.

## 3. Results

**Figure 1:**
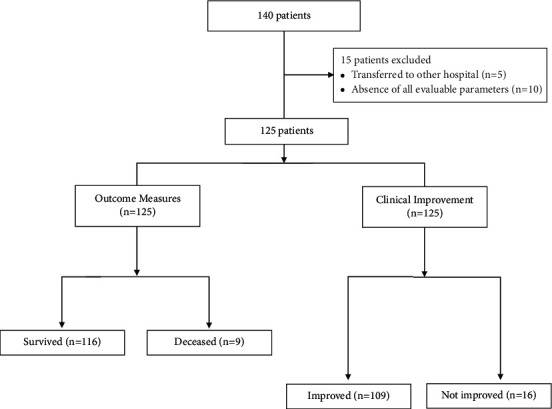
Patient inclusion and outcome.

A total of 140 patients with COVID-19 pneumonia were identified who received TCZ between May 2020 and August 2021. Among them, the data related to 125 patients were analyzed after excluding 15 patients (Figure 1). For analysis, patients were divided into two groups based on survival (survived and deceased) and clinical improvement (improved and not improved). Among 125 patients who received TCZ, 9 patients died (7.2%). The mean time to death was 22.2 ± 14.8 (4–52) days after administration of TCZ. A total of 109 patients improved (87.2%) by day 28 using the six-category ordinal scale.

The baseline characteristics of patients on the day of receiving TCZ are presented in Table 1. Patients who survived were significantly younger (47.25 ± 12.53) compared to patients who died (60.89 ± 10.54) (*P*=0.002). Similarly, patients who improved were also significantly younger (46.59 ± 12.33) compared to patients who did not improve (59.44 ± 10.83) (*P* ≤ 0.001).

Patients who survived had 9.19 ± 3.17 days to TCZ administration from the onset of symptoms, which is significantly shorter (*P*=0.036) than those who died (11.56 ± 3.9). Furthermore, patients who survived had CRP levels of 115.7 ± 69.34, which was significantly higher (*P*=0.028) than those who died (63.4 ± 40.91).

A lower ANC of 8617 ± 4668 was seen among patients who survived compared to 9223 ± 2287 in those who did not survive (*P*=0.017). Patients who were intubated at the time of administration of TCZ had a higher risk of death (RR = 10.46; 95% CI = 1.52–75.2; *P*=0.041).

13 survived patients out of 116 were vaccinated (11.2%), and 1 dead patient out of 9 was vaccinated (11.1%). This shows no difference in mortality in patients who were vaccinated. On the other hand, 12/109 (11%) of improved patients were vaccinated compared to 3/16 (18.7%) in the not improved group.

Among the deceased, there was a higher proportion of patients with CVD (4/9 vs 6/116; *P*=0.002; RR = 14.66 {3.11–69.13}) and renal insufficiency (2/9 vs. 1/116; *P*=0.013; RR = 32.8 {2.62–407.8}). A similar trend of comorbidities was seen among patients who did not improve (Table 2).

Among the 125 patients included in the analysis, 20 patients received a second dose of TCZ due to clinical worsening as judged by the primary care team. A subanalysis of these 20 patients revealed that 11 (55%) improved by day 28 and 9 (45%) did not improve (*P*=0.005). Out of these 9 patients, 5 (20%) died (*P*=0.005).

Outcomes at day 28 are summarized in Table 3. Clinical improvement was achieved in 109 patients who received TCZ. On the day of administration of TCZ (day 1), 55.2% of patients received NIV or high-flow supplemental oxygen, and 38.4% of them received low-flow oxygen to manage hypoxia. By day 14, 52.8% of patients were discharged from the hospital, 9.6% were mechanically ventilated, and 1.6% of patients were deceased.

**Figure 2:**
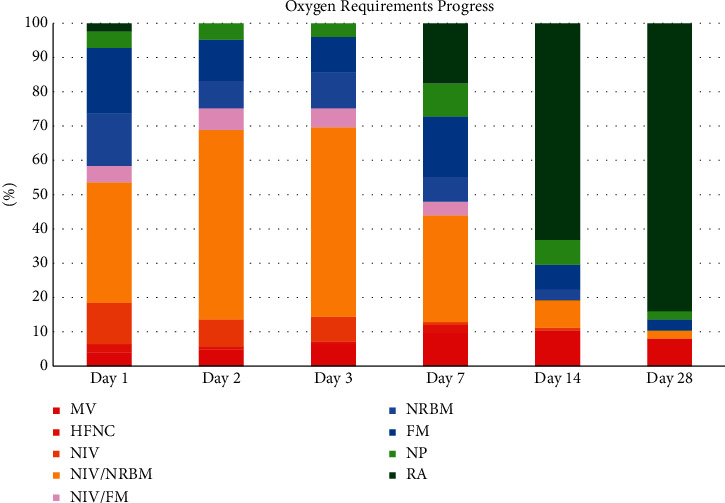
Oxygen requirements in patients with TCZ.

By day 28, a majority of them (80%) were discharged, and 7 patients (5.6%) were dead. Among those who were still hospitalized, 7.2% were mechanically ventilated, 3.2% did not require supplemental oxygen, 2.4% were on NIV or high-flow oxygen, and 1.6% were required low-flow oxygen. The progression of oxygen requirements is shown in Figure 2.

**Figure 3:**
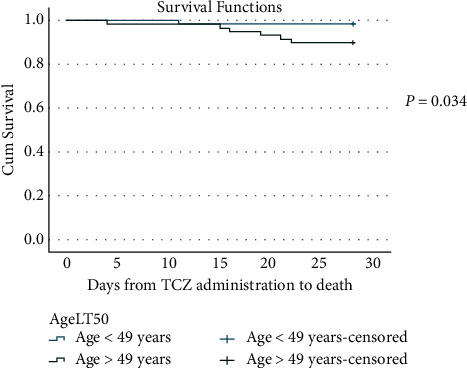
Kaplan–Meier curve for survival.

The survival curve calculated with the Kaplan–Meier method is plotted in Figure 3 (log rank, *P*=0.034). The 28-day cumulative survival was 98.55% (67/68) among those who were less than 49 years of age compared to 89.8% (53/59) in patients above 49 years of age.

## 4. Discussion

Analysis of risk factors of survival and clinical improvement among patients who received TCZ hospitalized for COVID-19 has shown significant findings. At the 4-week follow-up (day 28), 7 patients died, of which 2 patients had died by day 14. At the same 4-week follow-up, 109 patients improved, and 16 patients did not improve. By day 28, 80% of patients who were given TCZ were discharged. This percentage of patients discharged is much higher than the proportion discharged on TCZ (56.5%) in a randomized controlled trial [22]. This difference may be due to the fact that our analysis represents the use of TCZ in real-time patients where some patients received TCZ early impending clinical deterioration.

In our analysis, patients who were mechanically ventilated at the receipt of TCZ had a significantly higher risk of death but not clinical improvement at day 28, which is contrary to the findings of Rosas et al. in their randomized controlled trial where mechanical ventilation at baseline was significantly associated with nonimprovement but not death [22].

There were a greater number of males than females in our cohort. However, gender was not associated with the risk of death, as seen in previous studies [16]. As patients with a severe form of the disease received TCZ, our finding of more males than females is confirming the existing literature of male gender being a risk factor for severe disease [23, 24].

On the other hand, in both survived and improved groups, younger patients had better outcomes than older patients. Our finding is similar to existing reports of younger age being associated with improved outcomes [25] and older age being associated with mortality [26]. Patients 50 years and above had a lower survival compared to those under 50 years of age.

There was no significant difference in the time interval from the onset of COVID-19 symptoms to the hospitalization in clinical improvement and survival in our patients. This finding is contrary to the report presented in the ESCMID Conference on Coronavirus Disease by researchers from the University of Southern California that a shorter time from symptom onset to hospitalization is associated with a more serious disease and mortality [27]. However, there was a significant difference between the interval from onset symptoms to TCZ administration among patients who survived and died. Patients who survived received TCZ 2.37 days earlier than those who died from the onset of symptoms similar to the finding of Gupta et al. who reported that early treatment with TCZ was associated with improved outcomes [28].This is a significant finding which is contrary to the finding of Toniati et al. who reported that patients, who worsened or died, received TCZ 2 days earlier than those who improved [29]. Patients who survived had a mean duration of 9 days from the onset of symptoms to the administration of TCZ compared to 12 days among the deceased. This may be due to the earlier administration of TCZ, leading to better outcomes. Patients who died may have received TCZ after irreversible organ injury occurred.

In our cohort, patients who survived had significantly higher CRP than patients who died, which is similar to the finding of Mariette et al. that the benefit of TCZ was seen in patients with CRP levels of higher than 15.0 mg/dL, but not if less than 15.0 mg/dL [30]. This finding of higher CRP for improved survival in TCZ recipients is contradictory to the finding in the overall population with COVID-19 [31].The lower CRP in deceased patients could be as these patients might have developed a late flare of illness after administration of TCZ, and additionally, these are the patients who also have chronic conditions such as CKD and diabetes which makes them immunologically compromised. We did a subgroup analysis of patients who are over 75 and less than 75 mg/dL of CRP but did not find any significant difference in mortality. This points to the observation that CRP is not a good indicator for the start of TCZ, and its administration can be started even in patients with lower CRP if there are signs of clinical deterioration.

In the patients who received TCZ, one of the markers of efficacy observed is the rapid reduction of CRP levels to normal in a few days. This was also observed in other laboratory markers but to a smaller degree.

NLR levels were significantly higher in both deceased and not improved groups. High NLR level at the administration of TCZ is, therefore, a significant risk factor for mortality in our population, which is consistent with a meta-analysis conducted by Li et al. among 34 studies with COVID-19 patients and was also reported by Ahsan et al. that high NLR level was a strong predictor of death among COVID-19 patients [32, 33].

There were significantly higher levels of ANC in patients who did not improve and died, making high ANC levels a risk factor for mortality in COVID-19 patients receiving TCZ. This is in line with studies that state that ANC levels are higher in critical and severe COVID-19 patients [34].

There was no significant difference between survival as well as clinical improvement between vaccinated and nonvaccinated cohorts. However, a subgroup analysis revealed that there was only one death among 14 patients who received the vaccination. It is important to note that the cohort included in our analysis are the patients who developed the severe form of the disease, and this nonsignificant difference in mortality shall not be considered as vaccine efficacy.

The most common comorbidity in our cohort was diabetes followed by hypertension and dyslipidemia. The presence of CVD was significantly higher in deceased and not improved patients. This shows that CVD is a significant risk factor for mortality in our population treated with TCZ which is consistent with the Italian study done by Toniati et al. in patients who received TCZ [29]. This finding is also consistent with the literature on overall COVID-19 patients irrespective of receiving TCZ [35].

Renal insufficiency was present in 0.8% of survived patients and 22.2% of deceased patients. Like CVD, renal insufficiency is a significant risk factor for mortality in COVID-19 patients treated with TCZ as reported by Toniati et al. among their patients on TCZ. All patients with renal insufficiency did not improve by day 28, suggesting that renal insufficiency is associated with death in patients with COVID-19 whether they receive TCZ or not.

We did not find any significant difference in the proportion of comorbidities such as hypertension, diabetes, obesity, smoking, and pulmonary disease among survived and deceased groups, which is a very significant finding because all meta-analyses among COVID-19 patients have reported these comorbidities as risk factors for death [36–38]. However, a study done among patients receiving TCZ has reported no significant difference in comorbidities such as hypertension, diabetes, and COPD among patients who improved or did not improve [29]. The results of our study and the one conducted by Toniati et al. point to the hypothesis that administration of TCZ improves the outcomes in COVID-19 patients with comorbidities such as hypertension, diabetes, obesity, and COPD, which must be studied further.

A little over one-tenth of patients (12.8%) received an additional dose of TCZ due to nonimprovement after the first dose, with the majority of them improving after the second dose. The number of patients who received the second dose in our study is lower than in the randomized controlled phase 3 trial of TCZ, where the second dose was administered to 22.1% of first dose TCZ recipients [22].

One of the strengths of our study is that we have presented the observational data of 125 cases treated with TCZ, which is the highest number of patients in any observational cohort at a single center to our knowledge. Our analysis presents the data of patients from 22 nationalities, mostly Asians and Arabs. We have not analyzed the vaccinated subgroup for the manufacturer, the number of vaccine doses, and time lapse after the vaccine in our cohort. We do not have the repeat CT findings of all patients, as the repeat CT was done only in patients whose clinical condition worsened.

## 5. Conclusion

Younger age and earlier administration of TCZ were associated with improved outcomes in patients with COVID-19. There was no significant difference in comorbidities such as diabetes, hypertension, obesity, and pulmonary disease among patients who survived and deceased.

## Figures and Tables

**Table 1:** Baseline univariate risk factors of survival and improvement in TCZ patients

	Survived (*n* = 116)	Deceased (*n* = 9)	*p* value	Improved (*n* = 109)	Not Improved (*n* = 16)	*p* value
Age (years)	47.25 ± 12.52	60.89 ± 10.54	0.002	46.59 ± 12.33	59.44 ± 10.83	<0.001
Sex: Male	93	5	0.1	88	10	0.111
PaO2 (mmHg)	74.60 ± 30.68	84.11 ± 25.77	0.373	75.91 ± 31.54	73.50 ± 23.62	0.773
SpO2 (%)	90.56 ± 7.06	90.22 ± 4.99	0.888	90.83 ± 7.06	88.56 ± 5.64	0.223
Days from onset to hospitalization	7.47 ± 2.95	7.67 ± 3.24	0.845	7.48 ± 2.96	7.50 ± 2.98	0.977
Days from onset to TCZ	9.19 ± 3.17	11.56 ± 3.90	0.036	9.29 ± 3.17	9.81 ± 3.97	0.556
CRP (mg/dl)	115.7 ± 69.34	63.40 ± 40.91	0.028	112.9 ± 68	105 ± 76.98	0.671
LDH (U/L)	364.1 ± 156.9	544.3 ± 142.5	0.054	361 ± 146.5	447.8 ± 233.6	0.125
D-dimer (ng/ml)	545.5 ± 819.1	1445 ± 1808	0.176	537.2 ± 839	1144 ± 1429	0.129
Ferritin (ng/ml)	605.6 ± 512.4	1600 ± 2322	0.235	604.1 ± 525.6	1175 ± 1775	0.22
ALT (U/L)	56.16 ± 47.80	57.22 ± 34.89	0.948	54.09 ± 46.74	70.81 ± 46.68	0.184
ANC (10^3^/*µ*l)	8617 ± 4668	9223 ± 2287	0.017	8477 ± 4497	9223 ± 4681	0.047
NLR	12.49 ± 10.68	21.54 ± 13.50	0.018	12.28 ± 10.58	19.01 ± 13.01	0.023
S.Cr (mg/dl)	0.9 ± 0.22	1 ± 0.41	0.539	0.9 ± 0.2	1.02 ± 0.39	0.237
Procalcitonin (ng/ml)	0.314 ± 0.876	0.33 ± 0.417	0.956	0.303 ± 0.894	0.398 ± 0.432	0.686
CT involvement (%)	54.59 ± 14.37	49.38 ± 21.98	0.343	53.64 ± 14.07	58.57 ± 20.32	0.248
NIV Status	63	3	0.305	57	9	0.796
Intubation Status	3	2	0.041	3	2	0.122
Vaccination Status	13	1	1.00	12	2	1.00

ALT: Alanine transaminase, ANC: Absolute Neutrophil Count, CRP: C-Reactive Protein, LDH: Lactate Dehydrogenase, NIV: Noninvasive Ventilation, NLR: Neutrophil-Lymphocyte Ratio, S.Cr: Serum Creatinine

**Table 2:** Comorbidities of patients receiving TCZ.

	Survived (*n* = 116)	Deceased (*n* = 9)	*P* value	Improved (*n* = 109)	Not improved (*n* = 16)	*P* value

Hypertension	41	6	0.79	38	9	0.165
Diabetes	48	7	0.42	46	9	0.419
Dyslipidemia	23	3	0.392	22	4	0.742
Pulmonary disease	9	1	0.54	8	2	0.615
Obesity	57	4	0.791	54	7	1.00
Cardiovascular disease	6	4	0.002	5	5	0.003
Renal insufficiency	1	2	0.013	0	3	0.002
Smoking	10	0	1.00	9	1	1.00
Others	26	6	0.009	23	9	0.005

**Table 3:** Change in clinical status in patients with TCZ.

Clinical status	Baseline TCZ (%)	Day 14(%)	Day 28(%)

1. Discharged from hospital	0	52.8	80
2. Hospitalization, not requiring supplemental O2	2.4	9.6	3.2
3. Hospitalization, requiring supplemental low-flow O2	38.4	13.6	1.6
4. Hospitalization, requiring NIV and/or high-flow supplemental O2	55.2	12.8	2.4
5. Hospitalization, requiring invasive mechanical ventilation or ECMO	4	9.6	7.2
6. Death	0	1.6	5.6

## Data Availability

Data are available on reasonable request. The deidentified participant datasets analyzed for this study are available on reasonable request from the corresponding author at dr.shabaz@gmu.ac.ae.

## References

[1] Ruiz-Antorán B., Sancho-López A., Sancho-López A. (2021). Combination of tocilizumab and steroids to improve mortality in patients with severe COVID-19 infection: a Spanish, multicenter, cohort study. *Infectious Disease and Therapy*.

[2] Sundarakani B. (2017). Transforming dubai logistics corridor into a global logistics hub. *Asian Journal of Management Cases*.

[3] Natasha T. *First Middle East Cases of Coronavirus Confirmed in the UAE CNBC.Com*.

[4] Staff R. (2021). UAE daily coronavirus cases surge to near peak level. https://www.reuters.com/article/us-health-coronavirus-emirates-idINKBN2622FU.

[5] (2020). UAE announces first two deaths from coronavirus: WAM. *Reuters*.

[6] Alsuwaidi A. R., Al Hosani F. I., ElGhazali G., Al-Ramadi B. K. (2021). The COVID-19 response in the United Arab Emirates: challenges and opportunities. *Nature Immunology*.

[7] National committee for Management of COVID-19 Cases (2020). *National Guidelines for Clinical Management and Treatment of COVID-19 March 19, 2020 Version 1.1*.

[8] National committee for Management of COVID-19 Cases (2020). *National Guidelines for Clinical Management and Treatment of COVID-19 April 3, 2020 Version 2.0*.

[9] Liu D., Zhang T., Wang Y., Xia L. (2020). Tocilizumab: the key to stop coronavirus disease 2019 (COVID-19)-Induced cytokine release syndrome (CRS)?. *Frontiers of Medicine*.

[10] Patel A., Shah K., Dharsandiya M. (2020). Safety and efficacy of tocilizumab in the treatment of severe acute respiratory syndrome coronavirus-2 pneumonia: a retrospective cohort study. *Indian Journal of Medical Microbiology*.

[11] Xu X., Han M., Li T. (2020). Effective treatment of severe COVID-19 patients with tocilizumab. *Proceedings of the National Academy of Sciences*.

[12] Luo P., Liu Y., Qiu L., Liu X., Liu D., Li J. (2020). Tocilizumab treatment in COVID-19: a single center experience. *Journal of Medical Virology*.

[13] Hamed D. M., Belhoul K. M., Al Maazmi N. A. (2021). Intravenous methylprednisolone with or without tocilizumab in patients with severe COVID-19 pneumonia requiring oxygen support: a prospective comparison. *Journal of Infection and Public Health*.

[14] Assiri A., Iqbal M. J., Mohammed A. (2021). COVID-19 related treatment and outcomes among COVID-19 ICU patients: a retrospective cohort study. *Journal of Infection and Public Health*.

[15] Desai H. D., Sharma K., Parikh A. (2021). Predictors of mortality AmongstTocilizumab administered COVID-19 asian Indians: a predictive study from a tertiary care centre. *Cureus*.

[16] Sarabia De Ardanaz L., Andreu-Ubero J. M., Navidad-Fuentes M. (2021). Tocilizumab in COVID-19: factors associated with mortality before and after treatment. *Frontiers in Pharmacology*.

[17] Cassone G., Dolci G., Besutti G. (2020). Acute-phase reactants during tocilizumab therapy for severe COVID-19 pneumonia. *Clinical & Experimental Rheumatology*.

[18] Tomazini B. M., Maia I. S., Cavalcanti A. B. (2020). Effect of dexamethasone on days alive and ventilator-free in patients with moderate or severe acute respiratory distress syndrome and COVID-19: the CoDEX randomized clinical trial. *JAMA*.

[19] Campochiaro C., Della-Torre E., Cavalli G. (2020). Efficacy and safety of tocilizumab in severe COVID-19 patients: a single-centre retrospective cohort study. *European Journal of Internal Medicine*.

[20] Hill J. A., Menon M. P., Dhanireddy S. (2021). Tocilizumab in hospitalized patients with COVID‐19: clinical outcomes, inflammatory marker kinetics, and safety. *Journal of Medical Virology*.

[21] WHO (2021). Coronavirus disease (COVID-2019) R&D. https://www.who.int/blueprint/priority-diseases/key-action/novel-coronavirus/en/.

[22] Rosas I. O., Bräu N., Waters M. (2021). Tocilizumab in hospitalized patients with severe covid-19 pneumonia. *New England Journal of Medicine*.

[23] Wild J. M., Porter J. C., Molyneaux P. L. (2021). Understanding the burden of interstitial lung disease post-COVID-19: the UK interstitial lung disease-long COVID Study (UKILD-Long COVID). *BMJ Open Respiratory Research*.

[24] Peckham H., de Gruijter N. M., Raine C. (2020). Male sex identified by global COVID-19 meta-analysis as a risk factor for death and ITU admission. *Nature Communications*.

[25] Patel M., Gangemi A., Marron R. (2020). Retrospective analysis of high flow nasal therapy in COVID-19-related moderate-to-severe hypoxaemic respiratory failure. *BMJ Open Respiratory Research*.

[26] Cheng D., Calderwood C., Skyllberg E., Ainley A. (2021). Clinical characteristics and outcomes of adult patients admitted with COVID-19 in East London: a retrospective cohort analysis. *BMJ Open Respiratory Research*.

[27] Emily Henderson B. (2020). Shorter time from COVID-19 symptom onset to hospitalization linked to disease severity, death. https://www.news-medical.net/news/20200926/Shorter-time-from-COVID-19-symptom-onset-to-hospitalization-linked-to-disease-severity-death.aspx.

[28] Gupta S., Wang W., Hayek S. S. (2021). Association between early treatment with tocilizumab and mortality among critically ill patients with COVID-19. *JAMA Internal Medicine*.

[29] Toniati P., Piva S., Cattalini M. (2020). Tocilizumab for the treatment of severe COVID-19 pneumonia with hyperinflammatory syndrome and acute respiratory failure: a single center study of 100 patients in Brescia, Italy. *Autoimmunity Reviews*.

[30] Mariette X., Hermine O., Tharaux P. L. (2021). Effectiveness of tocilizumab in patients hospitalized with COVID-19: a follow-up of the CORIMUNO-TOCI-1 randomized clinical trial. *JAMA Internal Medicine*.

[31] Izcovich A., Ragusa M. A., Tortosa F. (2020). Prognostic factors for severity and mortality in patients infected with COVID-19: a systematic review. *PLoS One*.

[32] Li Y., Hou H., Diao J., Wang Y., Yang H. (2021). Neutrophil-to-lymphocyte ratio is independently associated with COVID-19 severity: an updated meta-analysis based on adjusted effect estimates. *The International Journal of Literary Humanities*.

[33] Ahsan T., Rani B., Siddiqui R. (2021). Clinical variants, characteristics, and outcomes among COVID-19 patients: a case series analysis at a tertiary care hospital in karachi, Pakistan. *Cureus*.

[34] Aly M. M., Meshref T. S., Abdelhameid M. A. (2021). Can hematological ratios predict outcome of COVID-19 patients? A multicentric study. *Journal of Blood Medicine*.

[35] Hessami A., Shamshirian A., Heydari K. (2021). Cardiovascular diseases burden in COVID-19: systematic review and meta-analysis. *The American Journal of Emergency Medicine*.

[36] Zheng Z., Peng F., Xu B. (2020). Risk factors of critical & mortal COVID-19 cases: a systematic literature review and meta-analysis. *Journal of Infection*.

[37] Longmore D. K., Miller J. E., Bekkering S. (2021). Diabetes and overweight/obesity are independent, nonadditive risk factors for in-hospital severity of COVID-19: an international, multicenter retrospective meta-analysis. *Diabetes Care*.

[38] Liu Y., Pan Y., Yin Y., Chen W., Li X. (2021). Association of dyslipidemia with the severity and mortality of coronavirus disease 2019 (COVID-19): a meta-analysis. *Virology Journal*.

